# P17 Hepatobiliary parasitic infestation: a case report

**DOI:** 10.1093/jacamr/dlad143.021

**Published:** 2024-01-03

**Authors:** Omar Al-Anee, Izhar Bagwan, Amila Gamage

**Affiliations:** Department of Cellular Pathology & Surgery, Royal Surrey Hospital NHS Foundation Trust; Department of Cellular Pathology & Surgery, Royal Surrey Hospital NHS Foundation Trust; Department of Cellular Pathology & Surgery, Royal Surrey Hospital NHS Foundation Trust

## Abstract

**Background:**

Hepatic parasitic lesions can present with multiple cystic lesions that may simulate inflammatory pseudo-tumour or malignancy in appropriate clinical settings. Here we present a known case of renal cell carcinoma with liver lesions that showed parasitic infestation on histology.

**Patient case:**

A 70-year-old male with a history of right partial nephrectomy for clear cell renal cell carcinoma having an uneventful postoperative follow-up for 2 years underwent a surveillance CT scan. Multiple hepatic cystic lesions were identified, which on subsequent MRI and PET scan were suspicious for metastasis. Partial hepatectomy was performed for histological evaluation.

**Results:**

Multiple discrete cystic hepatic lesions were noted on macroscopy. Histological examination revealed multiple necrotic hyalinized nodules with granulomatous inflammation within the liver and dense neutrophil infiltration with microabscess formation. Within the central necrotic area, there were non-viable parasite eggs and parts of worm, in keeping with parasitic infestation. On further expert opinion, this was diagnosed as *Enterobius* infestation of liver.

**Conclusions:**

Hepatobiliary parasitic infestations may be encountered as clinically and biochemically asymptomatic lesions, especially in immunocompromised patients. Strong clinical history of travel and parasite serology screen is always helpful to clinch the diagnosis but may not be always available during histological examination. This case highlights the importance of thorough histological and microbiological examination as all nodular lesions in liver are not malignant and could be infective.

